# Study on Regulatory Mechanism of *Gastrodia elata* Specific microRNA Targeting JNK3 in Alzheimer’s Disease

**DOI:** 10.3390/molecules31122075

**Published:** 2026-06-12

**Authors:** Hongyao Li, Zhongteng Lu, Ke Gao, Jianjun Guo, Zuoming Nie, Qing Sheng

**Affiliations:** 1 College of Life Sciences and Medicine, Zhejiang Sci-Tech University, Hangzhou 310018, China; 2023210901033@mails.zstu.edu.cn (H.L.); 202020801051@mails.zstu.edu.cn (Z.L.); 2022332864026@mails.zstu.edu.cn (K.G.); biojjguo@zstu.edu.cn (J.G.); wuxinzm@zstu.edu.cn (Z.N.); 2 Shaoxing Academy of Biomedicine, Zhejiang Sci-Tech University, Shaoxing 312000, China

**Keywords:** Alzheimer’s disease, *Gastrodia elata* miRNA, cross-kingdom, JNK3, tau hyperphosphorylation

## Abstract

Alzheimer’s disease (AD) is characterized by Tau hyperphosphorylation, β-amyloid (Aβ) accumulation, and progressive neuronal loss. *Gastrodia elata* (*G. elata*), a traditional Chinese medicine with well-established neuroprotective properties, was investigated. Two *G. elata*-derived miRNAs, Gas-miR04-3p and Gas-miR19-5p, were identified as regulators of JNK3. By means of Western blot, RT-qPCR, and assessments of antioxidant indices, it was demonstrated that Gas-miR04-3p and Gas-miR19-5p can suppress JNK3 expression, reduce Tau phosphorylation at Ser202 and Ser396, enhance antioxidant capacity, and attenuate apoptosis in AD-related cellular and molecular pathology models. These miRNAs were also detectable in murine brain tissues following oral administration of total RNA extracted from *G. elata*. Their administration was associated with decreased JNK3 activation, alleviated Tau hyperphosphorylation, and improved expression of apoptosis-related proteins in AD mouse models. These results suggest that *G. elata* miRNAs may exert neuroprotective effects through regulation of JNK3 signaling, thereby attenuating Tau-related pathological changes and neuronal injury in AD-related models.

## 1. Introduction

Alzheimer’s disease (AD), the most common cause of dementia and one of the major neurodegenerative diseases affecting the elderly population, is a degenerative disease of the central nervous system (CNS), first discovered and named by Alois Alzheimer in 1906 [1]. The disease is insidious and not easy to detect, and clinically patients mainly show different degrees of memory loss, cognitive and language comprehension disorders, etc., making it part of a class of diseases affecting the quality of human survival [2]. Globally, approximately 50 million people live with AD, and this figure is projected to reach 152 million by 2050, driven by population aging. In China, the prevalence of AD and related dementias (ADRDs) has increased by 249.1% since 1990, with about 16.99 million cases in 2021, imposing a staggering burden on families and the healthcare system [3]. The total societal costs of AD, including direct medical expenses and informal caregiving, are substantial and rise sharply with disease severity. Recent evidence demonstrated that the overall socioeconomic burden of AD increases significantly with disease progression, with the total cost rising by approximately 1.5 times from mild to severe stages [4]. The pathogenesis of AD is complex and involves multiple proposed mechanisms. The most classic pathological features are now generally recognized as senile plaques (SPs) formed by extracellular deposition of β-amyloid protein (Aβ) and neurofibrillary tangles (NFTs) formed by intracellular hyperphosphorylation of Tau protein [5,6]. In addition, the oxidative stress serves as an important factor in neuronal injury [7]. SPs and NFTs induce the production of large amounts of reactive oxygen species (ROS) in vivo, oxidative stress occurs, and this exacerbates the development of AD [8]. Previous studies have shown that natural products with antioxidant activity can alleviate Aβ-induced oxidative injury and exert neuroprotective effects in neuronal cells [9]. The occurrence of AD is associated with a variety of cellular dysfunctions, including abnormal miRNA regulation, mitochondrial damage, neuroinflammation, cerebral ischemia and hypoxia, lipid metabolism and energy metabolism disorders, and other abnormal pathological manifestations that are also an important cause of the induction or exacerbation of AD [10,11]. Currently, the pharmacological treatment of AD mainly includes cholinesterase inhibitors, such as donepezil and rivastigmine, and the NMDA receptor antagonist memantine. These drugs can provide symptomatic relief by improving cognitive function or delaying behavioral decline; however, they do not alter the underlying pathophysiology of the disease and therefore cannot halt or reverse disease progression [12,13]. This has led to an increasing number of studies focusing on novel targets such as neuroinflammation, oxidative stress and pathological protein aggregation, with a view to discovering more effective therapeutic strategies.

c-Jun N-terminal kinase 3 (JNK3), also known in mammals as stress-activated protein kinase, belongs to a subfamily of Mitogen-activated protein kinases that can be activated by stimuli such as UV, amyloid exposure, growth factors, heat shock, oxidative stress, etc. [14,15,16,17]. Three *jnk* genes have been identified, *jnk1*, *jnk2* and *jnk3*, and different JNK isoforms have different substrate affinities [18]. JNK1 and JNK2 isoforms are widely distributed in most tissues, and JNK3 isoforms are predominantly expressed specifically in CNS neurons [19]. Normal physiological levels of activated JNK3 have a role in neuronal cell development and growth, but sustained activation of JNK3 leads to synaptic dysfunction and even apoptosis, resulting in memory deficits and neurodegeneration, which ultimately leads to neurodegenerative diseases such as AD and Parkinson’s disease [20,21,22]. Numerous studies have shown that JNK3 is important in neuronal apoptosis, oxidative stress, Tau protein hyperphosphorylation, and mitochondrial dysfunction. Therefore, it is important to explore JNK3 as a therapeutic target [23].

Tau proteins play a crucial role in stabilizing the neuronal cytoskeleton through their interaction with microtubule monomers. In addition, they are involved in multiple intra- and extracellular signaling pathways, making them essential for maintaining neuronal integrity and function [24]. According to existing studies, JNK3 overactivation increases phosphorylation of the serine/threonine sites of Tau proteins. Phosphorylation of both Ser202/Ser262 and Ser396/404 sites of Tau proteins in the brain can be mediated by JNK3 [25], and hyperactivation of the Ser202 site was strongly associated with the formation of NFTs [26]. Inhibition of JNK3 activity reduces the hyperphosphorylation of Tau protein and exerts neuroprotective effects [27,28]. Under normal physiological conditions, multiple metabolic pathways exist in the brain to maintain a certain level of Aβ [29]. However, in pathological conditions, the dynamic balance of Aβ production and degradation in the brain is disrupted, leading to an abnormal increase in Aβ as well as impaired clearance pathways, resulting in Aβ accumulation and promoting the development of AD [30]. JNK3 also induces conformational changes in APP by promoting phosphorylation of the Thr668 site at the carbonyl terminus of the APP protein to increase Aβ production [31], and mainly SP-insoluble components Aβ_42_, and it also induces JNK3 activation and promotes hyperphosphorylation of Tau protein [32,33,34]. In addition, JNK3 increases β-secretase expression by inducing hypoxia-inducible factor-1α expression, which further leads to Aβ accumulation [35,36]. Reducing the activation of JNK3 may somewhat reduce the accumulation of Aβ_42_ in the brain and maintain the balance between synthesis and metabolism.

MicroRNAs (miRNAs) are non-coding single-stranded RNA molecules consisting of 18–22 nucleotides that are widely found in the activities of living individuals; miRNAs degrade target mRNAs or inhibit their translation and regulate gene expression at the transcriptional or post-transcriptional level [37,38]. miRNAs are transcribed as primary transcripts and processed by Drosha into precursor miRNAs. After nuclear export, Dicer cleaves them into mature miRNAs, which are loaded into the RNA-induced silencing complex to regulate target mRNA expression via imperfect base pairing [39,40]. Endogenous miRNAs have many targets, and differential expression ultimately affects the expression of different genes, leading to different pathologic features [41,42]. The overactivation of the JNK3 pathway observed in AD may be associated with the differential expression of endogenous miRNAs. Many studies have pointed out that miRNAs play key regulatory roles in nervous system development and neurodegenerative diseases [43]. Both endogenous and exogenous miRNAs can inhibit Tau hyperphosphorylation by regulating target genes, offering potential strategies for the regulation of AD-related pathological processes. JNK3 is different from JNK1 and JNK2, and researchers have judged JNK3 to be a very promising target for AD therapy based on existing experiments [23]. Wang et al. [44] found that miR-335-5p could target and down-regulate JNK3 protein, alleviate Aβ accumulation and inhibit neuronal apoptosis, and they used mouse experiments to demonstrate that overexpression of miR-335-5p improves cognitive ability in mice, suggesting a role for JNK3 in the pathogenesis of AD.

Many traditional Chinese medicines and their extracts have demonstrated notable therapeutic effects in the treatment of various diseases [45,46]. *G. elata* is a highly specialized orchid plant whose dried tubers have been used since ancient times as a herbal medicine, with more applications in the treatment of vertigo, headache, cerebrovascular diseases, and neurodegenerative diseases [47,48,49]. As a perennial chlorophyll-lacking saprophytic orchid, *G. elata* is mainly distributed in mountainous forest areas of southwest, central and eastern China, and has been widely artificially cultivated to meet medicinal market demand. In addition to gastrodin and parishin E, *G. elata* contains a variety of bioactive constituents including parishin A/B, vanillyl alcohol, phenolics, polysaccharides and sterols, which collectively endow this herb with multiple pharmacological properties such as neuroprotection, antioxidation and anti-inflammation [50]. Gastrodin (Gas), the main active ingredient in *G. elata*, is a natural phenolic substance with sedative, antioxidant and anti-inflammatory effects [51,52,53]. It is a compound component that is more promising for research in neurodegenerative diseases such as AD [54]. More and more studies have found that miRNAs have important regulatory roles in diseases, and the expression of miRNAs shows some differences in pathological conditions, with overexpression or underexpression affecting the expression of related proteins [55,56]. Recent evidence has also suggested that certain plant-derived miRNAs may exert neuroprotective effects in AD-related models through the regulation of signaling pathways associated with Tau phosphorylation and neuronal injury [57].

In this research, we aim to investigate the potential protective mechanisms against AD by targeting the JNK3 signaling pathway through *G. elata* miRNAs on the basis of our previous work. *G. elata* was selected as the research material owing to its well-recognized neuroprotective advantages; compared with synthetic miRNAs and miRNAs from other plant sources, its endogenous miRNAs are natural, have low-toxicity, and possess specific targeting potency toward the AD key molecule JNK3. Specifically, we seek to determine whether *G. elata* miRNAs can directly target JNK3. This study will provide new insights into the molecular mechanisms underlying the neuroprotective effects of traditional Chinese medicine in AD-related pathology and offer a novel approach for the development and application of *G. elata*.

## 2. Results

### 2.1. Gas-miR04-3p and Gas-miR19-5p Regulate JNK3 Expression by Directly Binding to the 3′ Untranslated Region

The *G. elata* miRNAs were analyzed against the JNK3 3′-UTR sequence using three bioinformatic prediction programs, including RNAhybrid, RNA22 and miRanda. Candidate miRNAs were screened based on prediction consistency, favorable binding energies, and the presence of predicted binding sites within the JNK3 3′-UTR region. Among the predicted candidates, Gas-miR04-3p and Gas-miR19-5p exhibited relatively high prediction confidence and were therefore selected for further analysis. Two potential binding sites were ultimately identified for each miRNA (Figure S1), and the corresponding binding energies are summarized in Table S1).

Four recombinant plasmids (pmiRGLO-JNK3-miR04-WT, pmiRGLO-JNK3-miR04-MUT, pmiRGLO-JNK3-miR19-WT, pmiRGLO-JNK3-miR19-MUT) containing wild-type (WT) and mutant (MUT) fragments of the JNK3 3′-UTR were successfully constructed. PCR amplification of WT-JNK3-miR04-3p and MUT-JNK3-miR04-3p fragments yielded single bands of approximately 362 bp, indicating successful amplification of the target fragments (Figure S2A). Similarly, PCR products of WT-JNK3-miR19-5p and MUT-JNK3-miR19-5p showed specific bands at approximately 394 bp (Figure S2C). Double restriction digestion yielded two distinct electrophoretic bands for each recombinant plasmid: a major band at approximately 7350 bp corresponding to the p-miRGLO vector, and a smaller band at either 362 bp or 394 bp representing the inserted fragments (Figure S2B,D). These results confirm the successful construction and insertion of the target sequences into the vector. Sequencing results showed that the insertion fragments were correctly sequenced and the mutation sites were consistent with the designed (Figure S3A,B).

**Figure 1:**
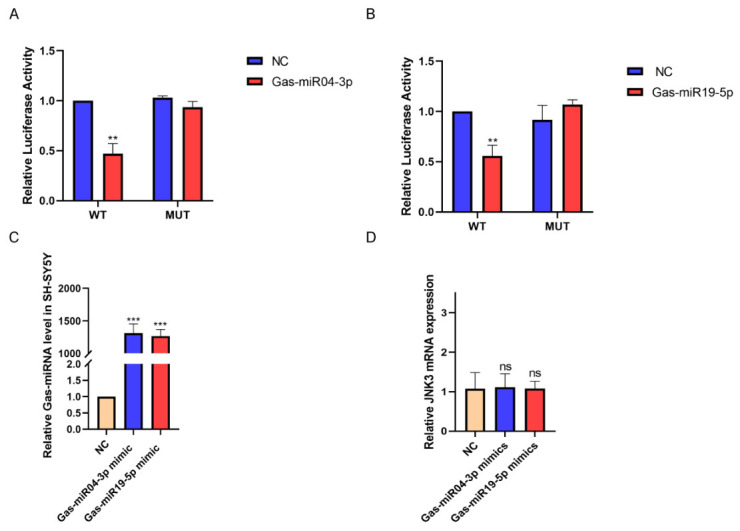
The relative luciferase activity A560/A465 and relative level of Gas-miRNA in SH-SY5Y cells. (**A**) Gas-miR04-3p; (**B**) Gas-miR19-5p; (**C**) relative level of Gas-miRNA in SH-SY5Y cells; (**D**) the relative levels of JNK3 mRNA in SH-SY5Y cells transfected with Gas-miRNA mimics. (ns, no statistical significance; ** *p* < 0.01; *** *p* < 0.001; vs. NC group).

Dual luciferase reporter assays revealed significantly reduced luciferase activity in the p-miRGLO-JNK3-miR04-WT group compared to the p-miRGLO-JNK3-miR04-MUT group, indicating that Gas-miR04-3p specifically binds to the JNK3 3′ -UTR of JNK3 and inhibits firefly luciferase expression (Figure S3A). Gas-miR19-5p exhibits similar effects (Figure S3B). These results are consistent with previous bioinformatics predictions and provide strong evidence that both Gas-miR04-3p (Figure 1A) and Gas-miR19-5p (Figure 1B) can target JNK3 mRNA 3′-UTR.

**Figure 2:**
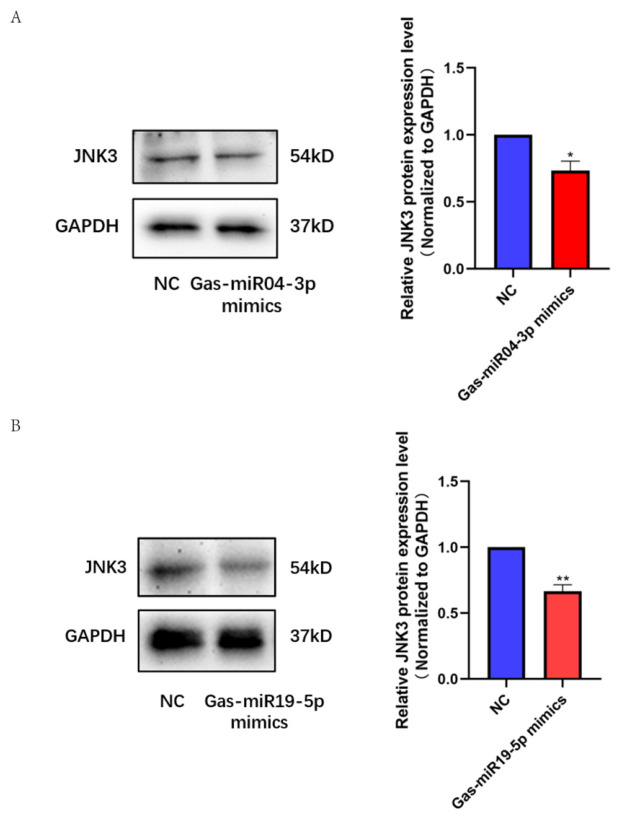
The protein level of JNK3 in SH-SY5Y cells transfected with Gas-miRNAs mimics. (**A**) Gas-miR04-3p; (**B**) Gas-miR19-5p. (* *p* < 0.05, ** *p* < 0.01, vs. NC group).

RT-qPCR results confirmed the successful transfection of Gas-miR04-3p and Gas-miR19-5p into cells (Figure 1C). However, no significant change in JNK3 mRNA expression levels was observed following treatment with either Gas-miR04-3p or Gas-miR19-5p (Figure 1D), and Western blot assay revealed that the protein expression level of JNK3 was down-regulated (Figure 2A,B), suggesting that Gas-miR04-3p and Gas-miR19-5p may affect JNK3 expression at the post-transcriptional level.

### 2.2. Oxidative Stress Indexes Show That Gas-miR04-3p and Gas-miR19-5p Mimics Can Exert Neuroprotective Effects in AD-Related Cellular Models

**Figure 3:**
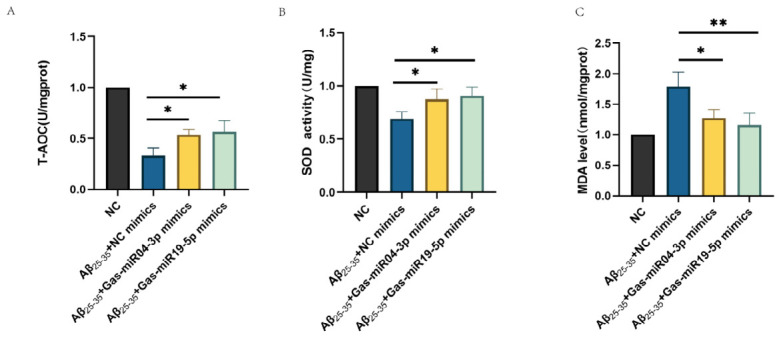
Effect of Gas-miRNA mimics treatment on oxidative stress index of AD cell model. (**A**) MDA level; (**B**) SOD activity; (**C**) T-AOC. (* *p* < 0.05, ** *p* < 0.01).

We transfected Gas-miR04-3p mimics and Gas-miR19-5p mimics respectively to treat AD cell models and assayed SOD/MDA/T-AOC indexes to explore their possible neuroprotective effects on AD cell models. In the AD model group, T-AOC and SOD levels decreased while MDA levels increased. After treatment with *G. elata* miRNA, T-AOC and SOD levels improved, and MDA levels decreased (Figure 3A–C). Following treatment with Gas-miR04-3p mimics and Gas-miR19-5p mimics, both *G. elata* miRNAs effectively reversed the AD model-induced reductions in T-AOC and SOD levels, as well as the increase in MDA content. These findings suggest that Gas-miR04-3p mimics and Gas-miR19-5p mimics exert certain neuroprotective effects on the AD cellular model.

### 2.3. Effect of G. elata miRNAs on Apoptosis in AD Cell Model

**Figure 4:**
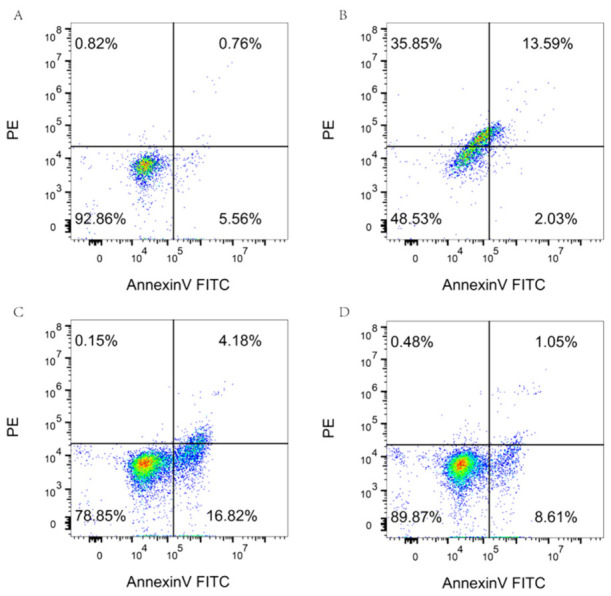
Effect on apoptosis of treatment with Gas-miRNA mimics. (**A**) NC; (**B**) Aβ_25–35_ + NC mimics; (**C**) Aβ_25–35_ + Gas-miR04-3p mimics; (**D**) Aβ_25–35_ + Gas-miR19-5p mimics.

**Figure 5:**
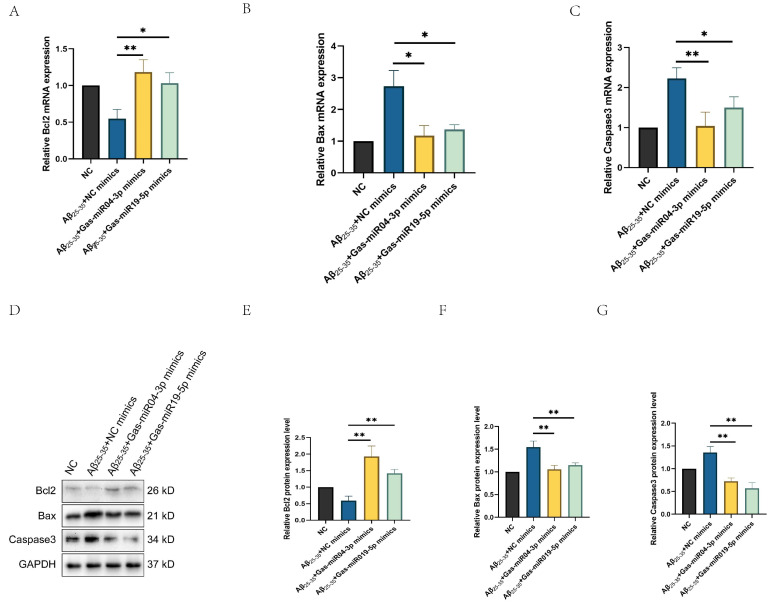
Effect of apoptosis-related gene and protein expression with Gas-miRNA mimics. (**A**) Bcl2 mRNA level; (**B**) Bax mRNA level; (**C**) Caspase3 mRNA level; (**D**) Western blot; (**E**) Bcl2 protein level; (**F**) Bax protein level; (**G**) Caspase3 protein level. (* *p* < 0.05, ** *p* < 0.01).

Gas-miR04-3p mimics and Gas-miR19-5p mimics were transfected and treated to explore the possible anti-apoptotic capacity of *G. elata* miRNAs. The results revealed that, compared with the NC group (Figure 4A), AD modeling induced a significant increase in apoptosis (Figure 4B), particularly elevating the rate of late-stage apoptosis. Treatment with Gas-miR04-3p mimics partially attenuated late apoptosis (Figure 4C); however, the early apoptosis rate remained elevated relative to the NC group. Notably, Gas-miR19-5p treatment (Figure 4D) exhibited the most prominent anti-apoptotic effect, markedly reducing both early- and late-stage apoptosis. These results suggest that Gas-miR04-3p mimics and Gas-miR19-5p mimics exert anti-apoptotic effects to varying degrees, with both effectively decreasing late-stage apoptosis. RT-qPCR analysis showed that AD induction down-regulated Bcl2 mRNA and up-regulated Bax and Caspase3 transcription. Treatment with Gas-miR04-3p or Gas-miR19-5p partially reversed these changes, and Western blot results demonstrated reduced Bcl2 and elevated Bax and Caspase3 protein levels in AD cells, which were ameliorated after miRNA treatment (Figure 5).

### 2.4. Gas-miR04-3p Mimics and Gas-miR19-5p Mimics Suppress Tau Phosphorylation in an AD Cell Model by Reducing the Activation of JNK3

**Figure 6:**
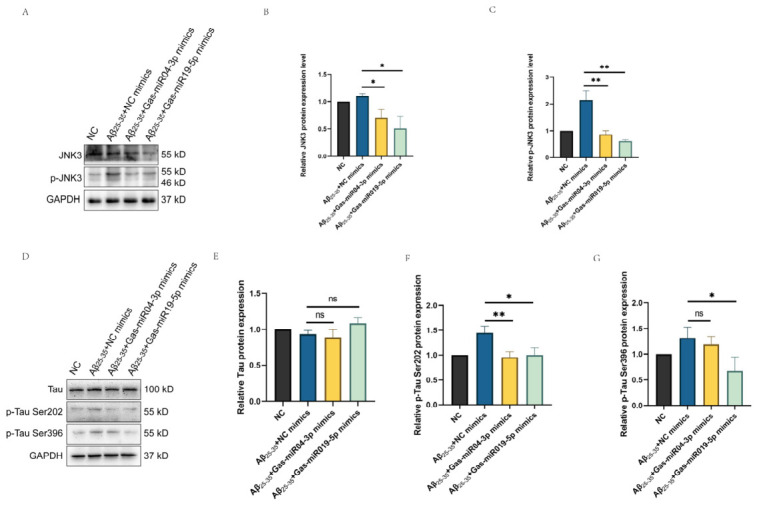
Effect of Gas-miRNA mimics on relative expression of JNK3 pathway-related protein and tau (Ser202) and tau (Ser396) in AD cell model. (**A**) Western blot; (**B**) JNK3 level; (**C**) p-JNK level; (**D**) Western blot; (**E**) Tau level; (**F**) p-Tau Ser202 level; (**G**) p-Tau Ser396 level. (ns, no statistical significance, * *p* < 0.05, ** *p* < 0.01).

The expression and phosphorylation of JNK3 proteins were assessed by Western blotting analysis after the AD cell models Gas-miR04-3p and Gas-miR19-5p were given treatments. The results showed that Aβ_25–35_ significantly increased the phosphorylation level of JNK3, whereas both miRNA mimics effectively inhibited the expression and decreased the phosphorylation level of JNK3 proteins (Figure 6A–C). Similarly, it was found that Aβ_25–35_ induced a significant increase in the phosphorylation levels of Tau protein at the Ser202 and Ser396 sites, while total Tau protein expression showed no significant difference among treatment groups (Figure 6E). In the Gas-miRNA mimic-treated groups, both Gas-miR04-3p and Gas-miR19-5p significantly reduced phosphorylation at the Ser202 site, and Gas-miR19-5p also effectively attenuated phosphorylation at the Ser396 site, though to a lesser extent (Figure 6F,G). These findings further demonstrate that, in vitro, Gas-miR04-3p and Gas-miR19-5p mimics may attenuate Tau protein hyperphosphorylation in AD cell models by inhibiting the JNK3 signaling pathway, thereby exerting neuroprotective effects in AD-related cellular models.

### 2.5. Effect of Total RNA Extract from G. elata on the Transcript Levels of JNK3 Pathway-Related Genes in the Mouse Brain

**Figure 7:**
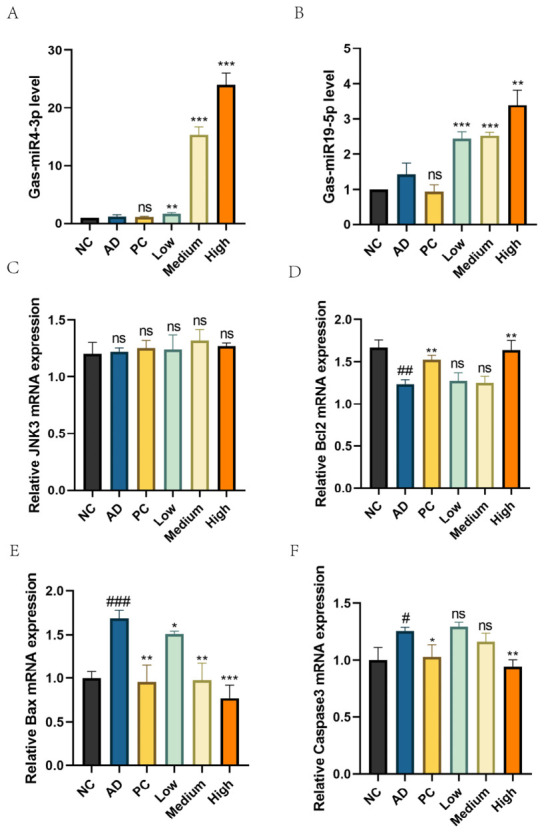
Relative level of Gas-miR04-3p, Gas-miR19-5p and JNK3-relatived signaling mRNA in mouse brain. (**A**) Gas-miR4-3p level; (**B**) Gas-miR19-5p level; (**C**) JNK3 level; (**D**) Bcl2 level; (**E**) Bax level; (**F**) Caspase3 level. (ns, no statistical significance, # *p* < 0.05, ## *p* < 0.01, ### *p* < 0.001 vs. NC group; * *p* < 0.05, ** *p* < 0.01, *** *p* < 0.001 vs. AD group).

After gavage of total RNA extracted from *G. elata*, both Gas-miR04-3p (Figure 7A) and Gas-miR19-5p (Figure 7B) were detected in the brain tissue of mice in a dose-dependent manner, with the highest levels detected in the high-dose group. These findings demonstrate that signals corresponding to Gas-miR04-3p and Gas-miR19-5p were detectable in mouse brain tissue following oral administration of total RNA extracted from *G. elata*, which is consistent with the possible presence of *G. elata*-derived miRNAs in brain tissue under the experimental conditions used in this study.

RT-qPCR was further utilized to detect the transcript levels of genes related to the JNK3 pathway (Figure 7C–F), and it was found that the levels of Bcl2 mRNA were decreased and the levels of Bax and Caspase3 mRNA were increased in the AD model mice, reflecting the onset of apoptosis in neuronal cells, while there was no significant change in JNK3 mRNA. In the donepezil hydrochloride group and the high-dose *G. elata* RNA group, the transcript level of Bcl2 was increased, and the transcript level of Bax and Caspase3 was decreased.

### 2.6. Regulation of JNK3 Signaling Pathway by Gas-miR04-3p and Gas-miR19-5p in AD Mouse Model

**Figure 8:**
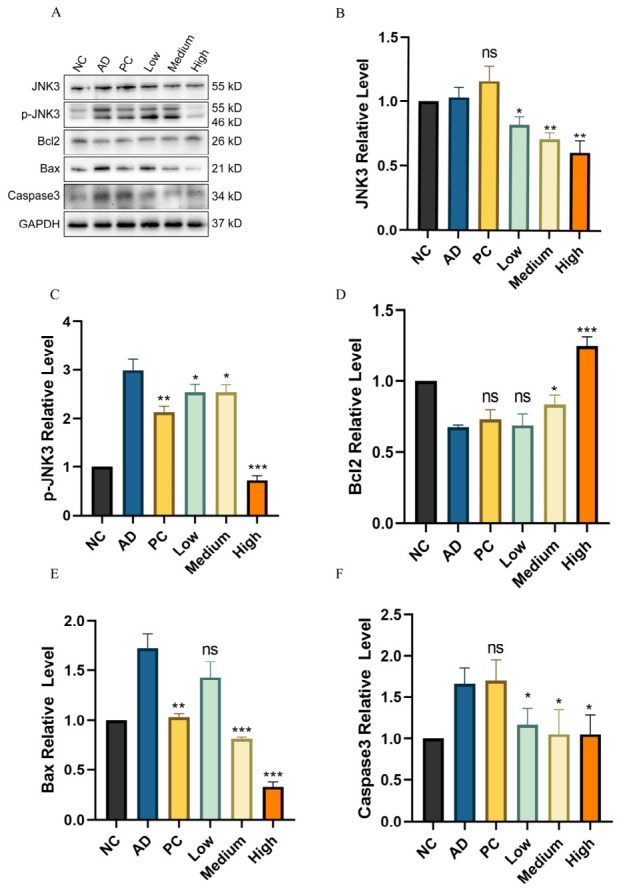
Relative expression level of JNK3 signaling and apoptosis-related proteins in mouse brain. (**A**) Western blot; (**B**) JNK3 level; (**C**) p-JNK3 level; (**D**) Bcl2 level; (**E**) Bax level; (**F**) Caspase3 level. (ns, no statistical significance, * *p* < 0.05, ** *p* < 0.01, *** *p* < 0.001; vs. AD group).

Further detection of JNK3 pathway-related protein expression in AD mice was carried out (Figure 8). The results showed that the level of p-JNK3 phosphorylation (Figure 8B,C) was significantly elevated in the AD model group, suggesting activation of the JNK3 pathway. The middle- and high-dose *G. elata* RNA groups and donepezil hydrochloride group were able to inhibit p-JNK3 activation, and the inhibition was the most obvious in the high-dose *G. elata* RNA group, which was hypothesized to exert a protective effect by targeting JNK3. The activation of the JNK3 pathway further promoted neuronal apoptosis, and Western blot assay showed that the expression of anti-apoptotic protein Bcl2 (Figure 8D) was down-regulated and the expression of pro-apoptotic proteins Bax (Figure 8E) and Caspase3 (Figure 8F) was up-regulated in the AD model group, suggesting that apoptosis occurred. High-dose *G. elata* RNA treatment up-regulated Bcl2, down-regulated Bax and Caspase3, and significantly improved the apoptotic status, while the donepezil hydrochloride group only down-regulated Bax to a certain extent, and had no significant effect on Bcl2 and Caspase3, suggesting that the effect of *G. elata* RNA was more comprehensive in regulating apoptosis-related pathways.

**Figure 9:**
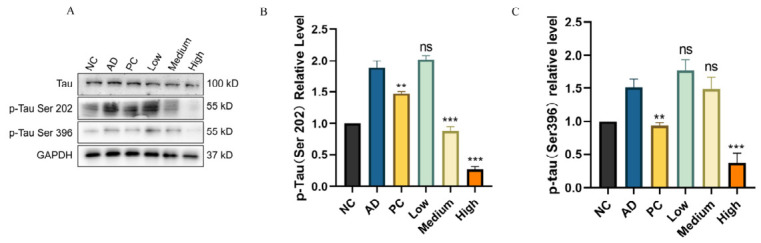
Relative expression level of Tau (Ser202 and Ser396) in mouse brain. (**A**) Western blot; (**B**) p-Tau Ser202 level; (**C**) p-Tau Ser396 level. (ns, no statistical significance, ** *p* < 0.01, *** *p* < 0.001; vs. AD group).

On the basis of the previous finding that high-dose *G. elata* RNA could inhibit the activation of the JNK3 pathway, we further detected Tau protein phosphorylation (Figure 9). The results showed that the phosphorylation levels of Tau protein Ser396 and Ser202 sites were elevated in AD model mice; whereas the phosphorylation of the Ser396 site was significantly reversed in the donepezil hydrochloride group and the high-dose *G. elata* RNA group, the Ser202 site was significantly decreased in the medium- and high-dose *G. elata* RNA groups and the donepezil hydrochloride group. This indicates that *G. elata* RNA can inhibit the abnormal phosphorylation of Tau protein at a certain dose and exert a neuroprotective effect similar to that of donepezil hydrochloride.

## 3. Discussion

AD is the leading type of dementia, and dementia has become a global health challenge, affecting an estimated 55.2 million people worldwide, according to the World Health Organization’s 2022 Blueprint for Dementia Research [58]. At this stage, more and more studies have demonstrated that endogenous miRNAs play regulatory roles in AD-related pathological processes by modulating key aspects such as inflammatory pathways, showing great promise as novel intervention targets [59]. Plant miRNAs play many key roles in the regulation of development, nutrient metabolism and adversity stress [60,61]. Plant miRNAs can substantially regulate protein synthesis and maintain developmental and metabolic homeostasis by complementary pairing with mRNAs, directing their cleavage or inhibiting translation, and playing a key role in the regulation of developmental and nutrient metabolism [62,63,64].

Notably, consistent with the typical post-transcriptional features of canonical miRNA regulation, we observed markedly decreased JNK3 protein levels but no significant alteration in JNK3 mRNA expression both in vitro and in vivo. This phenomenon strongly confirms that the two *G. elata*-derived miRNAs exert regulatory effects mainly through inhibiting the translational process of JNK3 mRNA, which is the most typical post-transcriptional regulatory mechanism of eukaryotic miRNAs. Mature miRNAs bind to the 3′-UTR of target mRNA through incomplete base pairing, which does not trigger mRNA degradation but effectively blocks ribosomal translation, thereby reducing protein synthesis without altering mRNA transcription level. This finding is highly consistent with the well-established regulatory mode of the miRNA-JNK3 signaling network in neurodegenerative diseases. Multiple recent studies have verified that numerous mammalian and exogenous miRNAs participate in AD pathological progression by targeting the 3′-UTR of JNK3 and inhibiting its protein translation, rather than affecting JNK3 mRNA stability, further supporting the reliability of our mechanistic results [44].

Existing studies have found that exogenous miRNAs of plant origin are stable in animals and are able to exert bioregulatory effects under cross-species conditions, and their potential functions in disease regulation and therapy are gaining attention [65]. Zhang et al. first demonstrated that rice-derived miR-168a can be absorbed into the murine circulatory system and regulate the expression of LDLRAP1 [66], thereby modulating cholesterol metabolism. Subsequently, honeysuckle-encoded miR-2911 was reported to directly inhibit influenza virus replication [67], grape-derived miR-159 was identified as being associated with breast cancer prognosis [68], and ginger-derived miR-167a was shown to modulate host inflammatory responses through extracellular vesicle-mediated transmission [69]. Plant miRNA-mediated cross-species regulatory mechanisms have also been widely discussed in plant–microbe interaction studies [70]. In the present study, the *G. elata* specific miRNAs Gas-miR04-3p and Gas-miR19-5p attenuate Tau hyperphosphorylation and neuronal injury by downregulating JNK3 expression and phosphorylation, thereby ameliorating key molecular pathological features in AD models. These findings enrich and extend the current body of evidence suggesting that plant-derived miRNAs may participate in cross-kingdom regulatory processes in mammals under certain experimental conditions. However, additional validation strategies, including stricter controls for contamination and sequencing confirmation, will still be required to further clarify the biological transport and distribution of these miRNAs in vivo.

*G. elata* has been traditionally used as a traditional Chinese medicine for the treatment of diseases related to the CNS [71]. Recent studies have shown that Parishin E, a phenolic compound isolated from *G. elata*, can significantly alleviate Aβ-induced toxicity in Caenorhabditis elegans models of AD by reducing Aβ aggregation and improving behavioral deficits [72]. This finding highlights the multifaceted neuroprotective potential of *G. elata*, not only through its small molecule components such as Parishin E, but also, as demonstrated in our study, through its endogenous miRNAs that modulate key pathological targets like JNK3. These results underscore the multi-component and multi-target therapeutic prospects of *G. elata* in the treatment of AD. The present study provides preliminary evidence that *G. elata*-derived miRNAs may participate in the regulation of JNK3-associated signaling pathways in AD-related pathological models. However, behavioral and cognitive assessments were not included in the present study. Therefore, whether these molecular and cellular changes are associated with improvements in learning and memory functions requires further investigation in future studies.

In the pathogenesis of AD, JNK3, as one of the isoforms of the JNK family, is thought to play a key role in neuronal stress injury and cell death due to its high expression specifically in the CNS [73,74]. It has been shown that JNK3 hyperactivation is closely associated with Aβ deposition, Tau protein phosphorylation, and neuroinflammation [75,76]. Transfection of Gas-miR04-3p mimics and Gas-miR19-5p mimics significantly down-regulated the expression of JNK3 protein and its phosphorylation level in AD model cells, which in turn inhibited the over-phosphorylation of Tau proteins and attenuated the occurrence of cell tangles. This aligns with accumulating evidence that cross-kingdom regulatory miRNAs from plants can modulate mammalian gene expression and exert biologically relevant outcomes.

Overall, these findings provide new mechanistic evidence that *G. elata* miRNAs suppress AD-related pathological cascades by targeting JNK3, thereby complementing the known effects of its bioactive small molecules. When considered alongside previous studies on cross-kingdom regulatory functions of plant miRNAs, this work highlights the therapeutic potential of medicinal plant encoded miRNAs as novel modulators of neurodegenerative disease processes. This integrated perspective underscores the multi-component and multi-target nature of traditional Chinese medicine and points to plant-derived miRNAs as an emerging frontier for AD intervention.

## 4. Materials and Methods

### 4.1. Cell Culture and Transfection

The SH-SY5Y cells were cultured in Dulbecco’s modified Eagle’s medium (DMEM, Gibco, Grand Island, NY, USA) supplemented with 10% fetal bovine serum (FBS, Gibco, Grand Island, NY, USA) in a humidified incubator of 5% CO_2_ and 95% air at 37 °C. SH-SY5Y cells were obtained from the Institute of America’s type culture collection (ATCC, Manassas, VA, USA). Gas-miR04-3p mimics (5′-AGGAAUGUUGUCUGGUUCGAA-3′), Gas-miR19-5p mimics (5′-CGGAGCUCUUCUUUCCCCACA-3′) and the negative control (NC) were synthesized by Gene Pharma (Shanghai, China) and dissolved in RNA-free H_2_O with a final concentration of 20 µM. Cells were inoculated onto various cell culture plates according to the instructions of the X-TremeGene siRNA transfection reagent (Roche, Basel, Switzerland). The transfection reagent was mixed with serum-free DMEM at different ratios and incubated at room temperature for 15 min before being added evenly to the cells. After transfection, the cells were further cultured for 24–48 h prior to subsequent experiments.

### 4.2. Animal Grouping and Treatment

Forty-eight C57BL/6 male mice weighing 22–25 g were obtained from Slycon (Shanghai, China). The fresh G. elata rhizomes were collected in November 2020 (winter) from Hanzhong, Shaanxi, China (geographical coordinates: N 33°04′, E 107°01′). All *G. elata* samples were collected at the same developmental stage with consistent growth status and sampling parts to ensure sampling uniformity and avoid the influence of tissue difference on miRNA expression profiles. The material was authenticated as *Gastrodia elata* Blume by experts from the Zhejiang Provincial Key Laboratory of Plant Secondary Metabolism Regulation. All animal handling and surgical operations were approved by the Animal Ethics Review Committee of Zhejiang Sci-Tech University.

Total RNA of fresh *G. elata* rhizomes was isolated using the classical TRIzol reagent method with unified and standardized experimental procedures [77]. Briefly, the rhizome samples were washed with PBS and fully lysed in TRIzol reagent, then incubated at room temperature for 5 min. Subsequently, 200 μL chloroform was added, mixed thoroughly and kept standing for 10 min at room temperature, followed by centrifugation at 12,000 rpm for 15 min at 4 °C. The upper aqueous phase was transferred to a new EP tube, mixed with an equal volume of isopropanol, and incubated for 10 min at room temperature. After centrifugation at 12,000 rpm for 10 min at 4 °C, the RNA precipitate was washed twice with 75% DEPC-treated ethanol, centrifuged at 6000 rpm for 5 min at 4 °C each time, air-dried at room temperature, and finally dissolved in RNase-free water.

C57BL/6 mice were randomly divided into a blank control NC group (Control), AD mouse model group (AD), positive drug control group (PC), *G. elata* RNA preparation low-dose group (Low), *G. elata* RNA preparation medium-dose group (Medium), and *G. elata* RNA preparation high-dose group (High), and 8 mice were taken from each group. Mice were given intraperitoneal injections of D-galactose (Solarbio, Beijing, China) at body weight (150 mg/kg) and gavage of AlCl_3_ (Solarbio, Beijing, China) at body weight (15 mg/kg) for 40 days. About 1h after modeling, the *G. elata* RNA was administered to the low-, medium- and high-dose groups by gavage at the different doses, which was maintained for 40 days. Total RNA of *G. elata* was administered to mice by gavage at low (3 mg/kg), medium (6 mg/kg), and high (9 mg/kg) doses according to their body weight. Before the formal experiment, mice were acclimatized to the laboratory environment for 7 days under standard housing conditions. Subsequently, D-galactose/AlCl3 modeling and *G. elata* RNA administration were continuously performed for 40 days. The mice were anesthetized using a gas anesthetic. Mice were sacrificed via exsanguination after eyeball blood collection and the mouse brain tissue was carefully removed.

### 4.3. RNA Extraction and Real-Time Quantitative PCR

Total RNA extraction from cell samples and mouse brain tissues was performed using the same TRIzol method and consistent experimental parameters as described for *G. elata* rhizome samples. Briefly, the samples were lysed with TRIzol reagent, separated by chloroform centrifugation, precipitated with isopropanol, and washed with 75% DEPC-treated ethanol to obtain purified total RNA. To minimize contamination and nonspecific amplification, all RNA extraction and RT-qPCR procedures were performed under RNase-free conditions using sterile consumables. Subsequently, reverse transcription was performed using a cDNA reverse transcription kit (Takara, Kyoto, Japan), and RT-qPCR was performed using the GoTaq^®^ qPCR and RT-qPCR Systems (Promega, Madison, WI, USA) kit with reverse-transcribed cDNA as a template. The primers used in this study were synthesized by Sangon (Shanghai, China) and are listed in Table S2. The reverse transcription process was as follows: the samples were incubated at 42 °C for 15 min, heat-shocked at 85 °C for 5 s, and then stored at 10 °C for a long time. The relative mRNA expression in each group was calculated by the ΔΔCT method based on the CT value of ABI 7500. Data results were analyzed and processed using GraphPad Prism 9.5 software.

### 4.4. Target Prediction and Dual-Luciferase Reporter Assay

The pre-obtained *G. elata* miRNAs were analyzed against the JNK3 3′-UTR sequences using three bioinformatic prediction programs, including RNAhybrid, RNA22 and miRanda. Candidate miRNAs were screened based on prediction consistency among the three algorithms, favorable minimum free energy values, and the presence of multiple predicted binding sites within the JNK3 3′-UTR region. Among the predicted candidates, Gas-miR04-3p and Gas-miR19-5p exhibited relatively high prediction confidence and were therefore selected for subsequent experimental validation. Subsequently, SH-SY5Y cellular DNA was used as a template to amplify fragments containing the predicted binding sites. Wild-type (WT) and mutant-type (MUT) insert fragments were constructed by subcloning and overlap PCR. The fragments were then ligated into the pmiRGLO dual-luciferase reporter vector to generate four recombinant plasmids: p-miRGLO-JNK3-miR04-WT, p-miRGLO-JNK3-miR04-MUT, p-miRGLO-JNK3-miR19-WT, and p-miRGLO-JNK3-miR19-MUT.

The correctness of plasmid construction was verified by colony PCR and double-enzyme digestion. The recombinant plasmids were subsequently cotransfected into SH-SY5Y cells together with NC mimics, Gas-miR04-3p mimics, or Gas-miR19-5p mimics, respectively. After 48 h, luciferase activities were measured using a dual-luciferase reporter assay kit (Gene Pharma, Shanghai, China), and fluorescence intensities at 560 nm and 465 nm were recorded for subsequent analysis.

### 4.5. Determination of Cellular Oxidative Stress Index

SH-SY5Y cells in logarithmic growth phase were inoculated in 6-well plates and transfected with *G. elata* miRNAs and NC mimics when the density reached 70%. The AD cell model was induced by adding 20 μM Aβ_25–35_ drug after 24 h. After cell treatment, cell samples were taken, washed with pre-cooled PBS three times and the supernatant was discarded. Cell lysate was added and lysed for 30 min at 4 °C. One portion of the sample was used for protein quantification by BCA and the other portion was processed according to the instructions of each kit. The readings were detected at the corresponding wavelengths of absorbed light using an enzyme marker and finally the values of T-AOC, SOD and MDA were calculated.

### 4.6. Western Blotting Analysis

After cell treatment, RIPA lysate (beyotime, Shanghai, China) containing PMSF (beyotime, Shanghai, China) and phosphatase inhibitor (cwbio, Beijing, China) was added, and the cells were lysed for protein quantification using the BCA kit (beyotime, Shanghai, China). The proteins were separated by SDS-PAGE electrophoresis, and protein transfer was performed by wet transfer method, where the separated proteins on the protein gel were transferred to a PVDF membrane, 100 V, 90 min. The primary antibodies were closed with 5% skimmed milk powder at room temperature for 2 h. The corresponding primary antibodies were incubated overnight at 4 °C with JNK3 (Abcam, Cambridge, UK, 1:1000), GAPDH (Proteintech, Chicago, IL, USA, 1:1000), total Tau (Proteintech, Chicago, IL, USA, 1:1000), p-Tau Ser 396 (Abcam, Cambridge, UK, 1:1000), and p-Tau ser 202 (Abcam, Cambridge, UK, 1:1000). On the following day, 1× TBST was washed 4 times for 5 min each time, and the incubation of secondary antibody, sheep anti-mouse IgG (Proteintech, Chicago, IL, USA, 1:5000), and sheep anti-rabbit IgG (Proteintech, Chicago, IL, USA, 1:5000) was continued for 1 h at 4 °C. After the end of 1× TBST wash 4 times, liquid A and B from ECL kit (beyotime, Shanghai, China), mixed 1:1, were configured as working solution, and uniformly added dropwise on the PVDF membrane, which was scanned and imaged by the Tanon 5500 Ultra-sensitive Chemiluminescence Instrument (Tanon, Shanghai, China). Results were processed using Image J 1.54t software and data were analyzed using Graphpad 8.3 software. Animal tissue samples were subjected to the same treatment and the volume of RIPA lysate was adjusted according to the tissue mass ratio.

### 4.7. Statistical Analysis

Data are presented as mean ± SEM from three independent biological experiments. Statistical analyses were performed using GraphPad Prism 8.3 software. Differences among multiple groups were analyzed using one-way ANOVA followed by Tukey’s multiple-comparison test. A *p* value < 0.05 was considered statistically significant.

## 5. Conclusions

As a traditional Chinese medicinal herb, *G. elata* has a long history of neuroprotective effects. In this study, two *G. elata* specific miRNAs (Gas-miR04-3p and Gas-miR19-5p) capable of targeting the 3′-UTR of JNK3 mRNA were predicted using bioinformatics methods, validated by dual-luciferase reporter gene assay, and Western blot and RT-qPCR assay identified JNK3 as the target gene of Gas-miR04-3p and Gas-miR19-5p.

In vitro experiments showed that transfection with Gas-miR04-3p and Gas-miR19-5p mimics significantly reduced AD-related cellular pathology, including decreased Tau phosphorylation, enhanced antioxidant capacity, and improved cell viability. These effects were associated with the downregulation of JNK3 expression and its phosphorylation activity. Additionally, the upregulation of anti-apoptotic proteins and downregulation of pro-apoptotic proteins led to reduced apoptosis, indicating that these miRNAs exert neuroprotective effects by modulating the JNK3 signaling pathway. To further validate these findings in vivo, Western blot analysis demonstrated that high-dose *G. elata* RNA treatment in mice similarly decreased JNK3 and phosphorylated Tau expression, while modulating apoptosis-related proteins in a manner consistent with the in vitro results. These results suggest that *G. elata* RNA may inhibit JNK3 pathway activation, attenuate Tau hyperphosphorylation, and reduce neuronal apoptosis, thereby potentially contributing to protective effects in AD-related pathological models. Notably, total RNA extracted from G. elata rather than individually purified Gas-miR04-3p or Gas-miR19-5p was administrated by intragastric gavage in the in vivo animal experiments. Therefore, the observed neuroprotective effects in AD model mice cannot be exclusively attributed to these two specific miRNAs, as numerous other endogenous RNAs and bioactive small molecules existing in total G. elata RNA may also participate in the in vivo regulatory effects. Further animal experiments with chemically synthesized pure Gas-miR04-3p and Gas-miR19-5p will be required to verify their independent in vivo functions in our follow-up work.

In recent years, accumulating studies have focused on cross-kingdom miRNA regulation, revealing that plant-derived miRNAs can cross biological boundaries and regulate gene expression in mammalian cells, which has become a research hotspot in the field of traditional Chinese medicine and neurodegenerative diseases [78,79]. Compared with previous reports, the present study provides preliminary evidence that *G. elata*-derived miRNAs may interact with mammalian JNK3-associated signaling pathways and participate in AD-related pathological regulation, which enriches our understanding of the molecular basis of cross-kingdom regulatory mechanisms of plant miRNAs.

In summary, this study investigated the regulation of the AD-associated JNK3 signaling pathway by Gas-miR04-3p and Gas-miR19-5p at cellular and animal levels, and preliminarily explored the potential molecular mechanism by which Gas-miR04-3p and Gas-miR19-5p may modulate JNK3 signaling and attenuate AD-related pathological changes, protein hyperphosphorylation, as well as the regulation of apoptosis-related factors. This study provides evidence supporting the neuroprotective effects of *G. elata*-derived miRNAs in AD-related pathological models. As novel bioactive components, plant endogenous miRNAs offer new insights into the pharmacological mechanisms of *G. elata* in neurodegenerative diseases such as Alzheimer’s disease, and this study will provide a theoretical reference for future studies on the regulation of AD-related pathological processes.

## Data Availability

The data that support the results of this study are available within the article and the Supplementary Materials. Further data are available from the corresponding author upon reasonable request.

## Supplementary Materials

The following supporting information can be downloaded at: https://www.mdpi.com/article/10.3390/molecules31122075/s1, Table S1: Binding of Gas-miR04-3p and Gas-miR19-5p to JNK3 mRNA and its minimum free energy by RNAhybrid; Table S2: Primer sequences used for reverse transcription and RT-qPCR. Figure S1: Target on JNK3 mRNA of Gas-miR04-3p and Gas-miR19-5p (A: Gas-miR19-5p complete binding site, B: Gas-miR19-5p miRNA 2-8 binding site and MUT site, C: Gas-miR04-3p complete binding site, D: Gas-miR04-3p miRNA 2-8 binding site and MUT site); Figure S2: Result of PCR and double digestion identification (A: Gas-miR04-3p PCR identification: M: 2k DNA Marker, 1: WT-JNK3-miR04, 2: MUT-JNK3-miR04; B: Gas-miR04-3p double digestion identification: M1: 10k DNA Marker, 1: WT-JNK3-miR04, 2: MUT-JNK3-miR04, M2: 2k DNA Marker; C: Gas-miR19-5p PCR identification: M: 2k DNA Marker, 1: WT-JNK3-miR19, 2: MUT-JNK3-miR19; D: Gas-miR19-5p double digestion identification: M1: 10k DNA Marker, 1: WT-JNK3-miR19, 2: MUT-JNK3-miR1, M2: 2k DNA Marker); Figure S3: Recombinant plasmid sequencing results (A: pmiRGLO-JNK3-miR04-WT, pmiRGLO-JNK3-miR04-MUT, B: pmiRGLO-miR19-JNK3-WT, pmiRGLO-miR19-MUT).
