# Supplementary material for: Study on Regulatory Mechanism of Gastrodia elata Specific microRNA Targeting JNK3 in Alzheimer’s Disease
Source: Molecules. 2026 Jun 12;31(12):2075. doi: 10.3390/molecules31122075 (PMC13306001; doi:10.3390/molecules31122075)
Supplement: Supplementary file 1 [file molecules-31-02075-s001.zip › Table S1.pdf]

Table S1. Binding of Gas-miR04-3p and Gas-miR19-5p to JNK3 mRNA and Its minimum free energy by RNAhybrid

MiRNA	mfe（Kcal/mol）
Gas-miR04-3p	-19.90
Gas-miR19-5p	-25.80
