# Supplementary material for: Study on Regulatory Mechanism of Gastrodia elata Specific microRNA Targeting JNK3 in Alzheimer’s Disease
Source: Molecules. 2026 Jun 12;31(12):2075. doi: 10.3390/molecules31122075 (PMC13306001; doi:10.3390/molecules31122075)
Supplement: Supplementary file 1 [file molecules-31-02075-s001.zip › Table S2.pdf]

Table S2. Primer sequences used for reverse transcription and RT-qPCR.

Primer name	Primer sequence（5′-3′）
JNK3 Forward	TATGCGGGACTCACCTTC
JNK3 Reverse	GGCGTCGTCCACTGATAT
GAPDH Forward	AATGGATTTGGACGCATTGGT
GAPDH Reverse	TTTGCACTGGTACGTGTTGAT
Gas-miR04-3p qPCR Forward	GCGGGAGGAATGTTGTCTGG
Gas-miR04-3p qPCR Reverse	CAGTGCAGGGTCCGAGGTAT
Gas-miR19-5p qPCR Forward	GCGGGCGGAGCTCTTCTTT
Gas-miR19-5p qPCR Reverse	CAGTGCAGGGTCCGAGGTAT
5.8s rRNA qPCR Forward	ATCACTCGGCTCGTGCGTC
5.8s rRNA qPCR Forward	CAAGTGCGTTCGAAGTGTCG
Gas-miR04-3p Loop-stem primer	GTCGTATCCAGTGCAGGGTCCGAGGTA<br>TTCGCACTGGATACGACTTCGAA
Gas-miR19-5p Loop-stem primer	GTCGTATCCAGTGCAGGGTCCGAGGTA<br>TTCGCACTGGATACGACACTGGA
