# Supplementary figures and images for: Study on Regulatory Mechanism of Gastrodia elata Specific microRNA Targeting JNK3 in Alzheimer’s Disease
Source: Molecules. 2026 Jun 12;31(12):2075. doi: 10.3390/molecules31122075 (PMC13306001; doi:10.3390/molecules31122075)

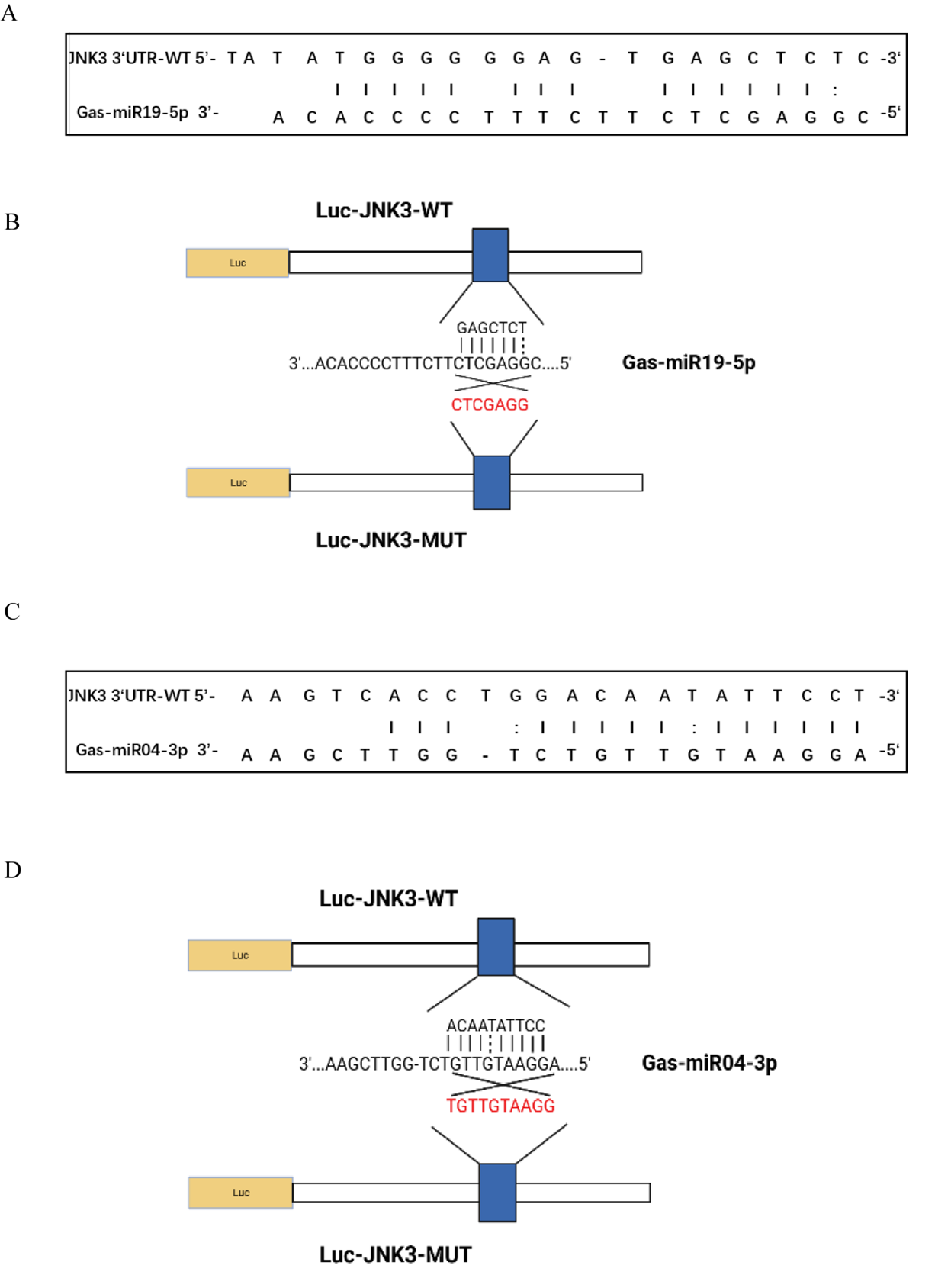

Supplement: Supplementary file 1 [file molecules-31-02075-s001.zip › Figure S1.tif]

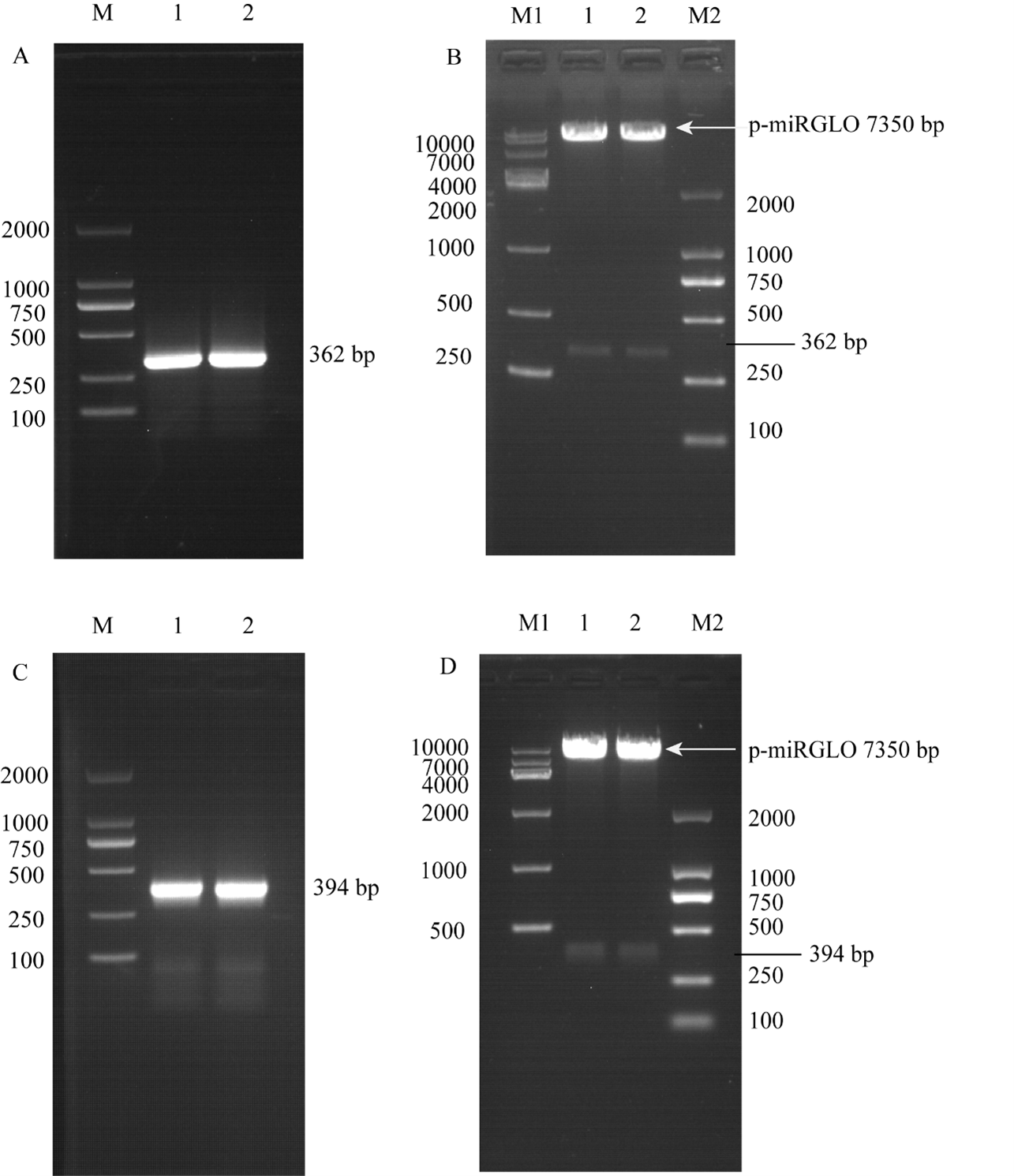

Supplement: Supplementary file 1 [file molecules-31-02075-s001.zip › Figure S2.tif]

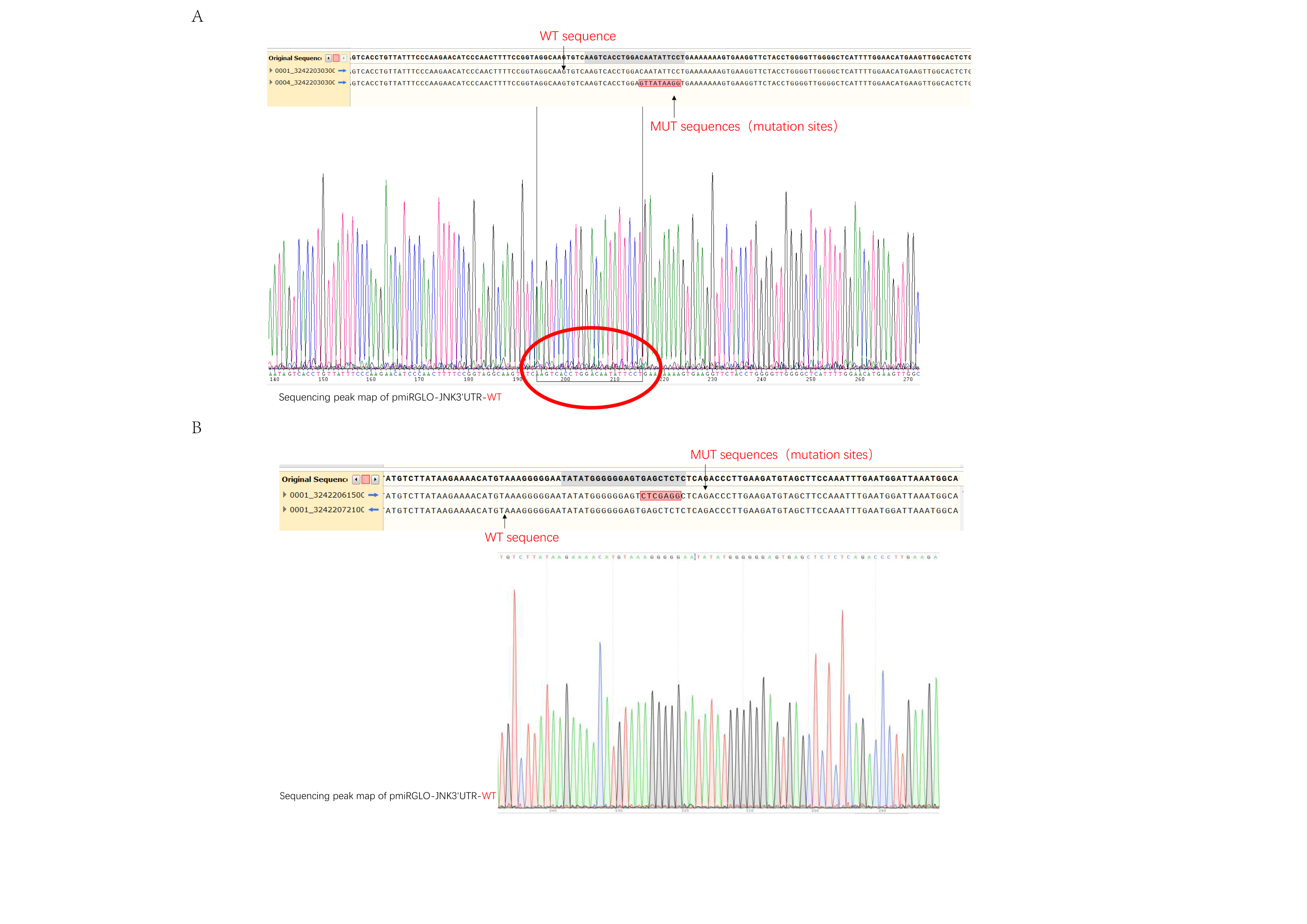

Supplement: Supplementary file 1 [file molecules-31-02075-s001.zip › Figure S3.tif]
